# Serum Uric Acid as a Cardiovascular Risk Marker: Differential Effects of Ketogenic Diet and Intermittent Fasting in Postmenopausal Women

**DOI:** 10.3390/nu18121912

**Published:** 2026-06-12

**Authors:** Barbara Pala, Laura Pennazzi, Mariagrazia Piscione, Giulia Nardoianni, Gemma Miletti, Serena De Mitri, Paola Gualtieri, Laura Di Renzo, Emanuele Barbato, Giuliano Tocci

**Affiliations:** 1 Division of Cardiology, Ospedale Istituto Dermopatico dell’Immacolata-Istituto di Ricovero e Cura a Carattere Scientifico (IDI-IRCCS), Via dei Monti di Creta, 104, 00167 Rome, Italy; 2 PhD School of Applied Medical-Surgical Sciences, University of Rome Tor Vergata, Via Montpellier 1, 00133 Rome, Italy; 3 Italian Society of Nutraceuticals (SINut), 40100 Bologna, Italy; 4 Fondazione Policlinico Campus Bio-Medico, University of Rome, Via Alvaro del Portillo, 200, 00128 Rome, Italy; 5 Division of Cardiology, Department of Clinical and Molecular Medicine, Faculty of Medicine and Psychology, University of Rome Sapienza, Sant’Andrea Hospital 1035/1039, 00189 Rome, Italy; 6 Section of Clinical Nutrition and Nutrigenomics, Department of Biomedicine and Prevention, University of Rome Tor Vergata, Via Montpellier 1, 00133 Rome, Italy; 7 Food Science Fellowship, University of Rome Tor Vergata, 00133 Rome, Italy

**Keywords:** serum uric acid, cardiovascular risk, URRAH, ketogenic diet, intermittent fasting, postmenopausal women, hypertension, metabolic risk, dietary intervention

## Abstract

Background: Serum uric acid (UA) is increasingly recognized as a cardiovascular (CV) risk factor, with evidence suggesting that CV risk may occur at levels below conventional hyperuricemia thresholds. However, real-world comparative data on the effects of dietary interventions on UA are limited. Objective: The primary objective of this study was to compare the effects of a very low-calorie ketogenic diet (VLCKD), intermittent fasting (IF), and standard dietary advice (free diet, FD) on UA levels and UA-defined CV risk in hypertensive postmenopausal women. Methods: In this prospective, single-center, real-world clinical study, 43 women with essential hypertension and obesity underwent dietary interventions. UA levels were assessed at baseline and after 6 months. Hyperuricemia was defined using both the conventional threshold (≥6.0 mg/dL) and the CV risk-oriented Uric Acid Right for Heart Health (URRAH) cut-off (≥4.7 mg/dL). Analyses included paired tests and ANCOVA adjusted for baseline UA. Results: At baseline, over half of participants exceeded the URRAH threshold. Only VLCKD significantly reduced UA levels (−1.23 mg/dL; *p* < 0.001), remaining independently associated after adjustment (β = −0.95 mg/dL; *p* = 0.007). URRAH-defined CV risk decreased significantly only in the VLCKD group (55.6% to 22.2%), with one-third transitioning to lower risk. Conclusions: VLCKD significantly reduced UA levels and UA-defined CV risk, supporting its role as a potentially effective lifestyle intervention in this population.

## 1. Introduction

Serum uric acid (UA) is the final product of purine metabolism in humans, who lack uricase and are therefore predisposed to higher circulating levels. UA homeostasis reflects the balance between production and excretion, with hyperuricemia resulting from increased synthesis, reduced renal or intestinal clearance, or both [1,2,3,4,5]. Dietary factors, including purine-rich foods, fructose intake, and alcohol consumption, together with metabolic conditions such as obesity, insulin resistance, metabolic syndrome, and hypertension, play a key role in modulating UA levels [6,7,8,9,10,11,12,13,14]. Beyond its metabolic role, UA has been implicated in oxidative stress, endothelial dysfunction, and vascular damage through mechanisms involving xanthine oxidase (XO) activity and reactive oxygen species (ROS) production [15,16,17,18,19].

Accumulating evidence indicates that UA is independently associated with cardiovascular (CV) risk, including hypertension, coronary artery disease, heart failure, and CV mortality [20]. Importantly, CV risk may increase at UA levels below the conventional hyperuricemia threshold. The Uric Acid Right for Heart Health (URRAH) study identified lower, sex-specific cut-off values associated with increased CV risk, particularly in women [21].

In recent years, growing interest has focused on the effects of dietary interventions such as the ketogenic diet (KD) and intermittent fasting (IF) on UA metabolism. Both approaches may induce an initial transient increase in UA due to renal competition mechanisms, followed by long-term reductions associated with weight loss, improved insulin sensitivity, and anti-inflammatory effects, including inhibition of the NLRP3 inflammasome [14,16,22,23,24,25,26,27].

However, despite these mechanistic insights, real-world comparative data on the impact of different dietary strategies on UA levels and UA-related CV risk remain limited, particularly in high-risk populations such as postmenopausal women with hypertension and obesity. Postmenopausal women represent a particularly relevant high-risk population for cardiometabolic disorders. Hormonal changes associated with menopause are linked to increased visceral adiposity, insulin resistance, and a higher prevalence of hypertension [28], all of which are known determinants of elevated serum UA levels and CV risk. In addition, previous evidence suggests that the relationship between UA and CV outcomes may be particularly pronounced in women, even at levels below conventional hyperuricemia thresholds.

Recent evidence further supports the role of UA–related biomarkers in CV risk stratification. In particular, composite indices such as the serum UA-to-creatinine ratio, as well as atherogenic indices, have been shown to independently predict adverse CV outcomes, including arrhythmia recurrence after catheter ablation [29]. Consistently, recent epidemiological and mechanistic evidence further strengthens the link between UA and CV risk. Large-scale analyses and dose–response meta-analyses have confirmed a significant association between serum UA levels and coronary heart disease risk [30]. In addition, population-based studies have shown that atherogenic lipid indices are closely associated with hyperuricemia, supporting a shared cardiometabolic risk profile [31]. Furthermore, emerging data highlight the role of dietary patterns and environmental factors in modulating UA metabolism, with high-purine and fructose-rich diets promoting hyperuricemia, whereas Dietary Approaches to Stop Hypertension (DASH) and Mediterranean dietary patterns are associated with lower UA levels and reduced cardiovascular risk [32,33]. Therefore, focusing on postmenopausal women provides a clinically meaningful framework to investigate the potential impact of dietary interventions on UA-related CV risk.

Based on these considerations, we hypothesized that different dietary strategies may exert distinct effects on serum UA levels and UA-related CV risk, with a potentially greater impact of a very-low-calorie ketogenic diet (VLCKD) compared with intermittent fasting (IF) and standard dietary advice [22]. The present study was designed as a real-world clinical investigation to evaluate the effects of a VLCKD, intermittent fasting (IF), and standard dietary advice on UA levels in hypertensive postmenopausal women with obesity. In addition, we assessed changes in UA-defined CV risk using both conventional thresholds and URRAH-derived cut-offs, with the aim of providing clinically relevant insights into the role of dietary interventions in CV risk modulation.

## 2. Materials and Methods

### 2.1. Study Design

The methodological framework of the parent study has been detailed elsewhere [22,23]. Briefly, between June 2022 and June 2023, we consecutively screened postmenopausal women referred to the Hypertension Unit, Division of Cardiology, Sant’Andrea Hospital, Rome (Italy) and at the Clinical Nutrition and Nutrigenomics Section, Tor Vergata University of Rome for hypertension screening including office and out-of-office blood pressure (BP) assessment, global CV risk evaluation, and assessment of hypertension-mediated organ damage (HMOD). Demographic and clinical characteristics were systematically collected at baseline.

#### 2.1.1. Inclusion and Exclusion Criteria

Inclusion criteria were: (1) female sex; (2) age between 50 and 65 years; (3) postmenopausal status; (4) diagnosis of overweight or obesity, as defined by body mass index (BMI) ≥ 25 kg/m^2^ [34]; (5) diagnosis of uncomplicated essential hypertension under stable antihypertensive therapy (excluding diuretics or losartan) for at least 6 months and without previous comorbidities, including previous myocardial infarction, acute coronary syndrome, transient ischaemic attack, stroke, acute or chronic kidney disease or congestive heart failure; (6) willingness to participate in clinical and nutritional follow-up, in accordance with the European Society of Hypertension guidelines for the management of hypertension (1). Exclusion criteria were any of the following ones: (1) uncontrolled or newly diagnosed diabetes mellitus, atrial fibrillation or major cardiac arrhythmias; (2) moderate-to-severe valvular heart disease; (3) acute CV events within the previous 6 months; (4) advanced chronic kidney disease or dialysis; (5) active or progressive oncological or systemic diseases, uncontrolled thyroid dysfunction, previous bariatric surgery or pharmacological treatments interfering with UA metabolism (including diuretics or losartan); (6) poor adherence to the dietary protocol.

#### 2.1.2. Outcomes

The investigation represents a predefined analysis within a previously described clinical protocol designed to evaluate the effects of different dietary interventions on cardiometabolic parameters in postmenopausal women with essential hypertension and overweight or obesity [35].

The primary outcome of the present analysis was the change in UA levels from baseline (T0) to 6 months (T6) in patients who underwent VLCKD or IF compared to those on Free Diet (FD). Secondary outcomes included the prevalence of hyperuricemia according to the conventional female-specific cut-off (≥6.0 mg/dL) and the URRAH CV risk–oriented threshold (≥4.7 mg/dL), and the association between UA levels and cardiometabolic parameters. As reported in the main study from the same database [22,23], the study protocol was submitted to the local Ethics Committee, which, on 14 July 2022, acknowledged the submission and did not issue any negative comments. The study was also registered on ClinicalTrial.gov (NCT06457711). All participants provided written informed consent prior to inclusion.

### 2.2. Anthropometric Measures

Anthropometric measurements were performed according to standardized procedures. Body weight was measured to the nearest 0.1 kg using a calibrated scale, with participants wearing light clothing and no shoes. Height was measured to the nearest 0.5 cm using a stadiometer, and body mass index (BMI) was calculated as weight (kg) divided by height squared (m^2^). BMI was classified according to World Health Organization criteria (overweight: 25.0–29.9 kg/m^2^; obesity: ≥30.0 kg/m^2^). Waist circumference was measured at the midpoint between the lower margin of the last rib and the iliac crest using a non-elastic tape, according to international recommendations. All measurements were obtained by trained personnel following standardized protocol, as previously reported [36].

### 2.3. Dietary Interventions

Upon enrolment, participants were allocated according to clinical and nutritional assessment to one of three dietary intervention groups for a total duration of six months, as specified in the main study protocol [22,23] (1) VLCKD, (2) IF, or (3) FD. The FD group represents standard-of-care dietary counseling in routine practice and was not intended as a controlled experimental comparator. Given the real-world design, dietary allocation was not randomized and reflected routine clinical decision-making after a shared clinician–patient discussion based on nutritional eligibility, contraindications, preferences, and expected adherence. Caloric intake was individualized but prescribed within standardized ranges to ensure comparability across dietary interventions. The main characteristics of the three dietary interventions are summarized in Table 1 to facilitate comparison between regimens.

**Figure 1:**
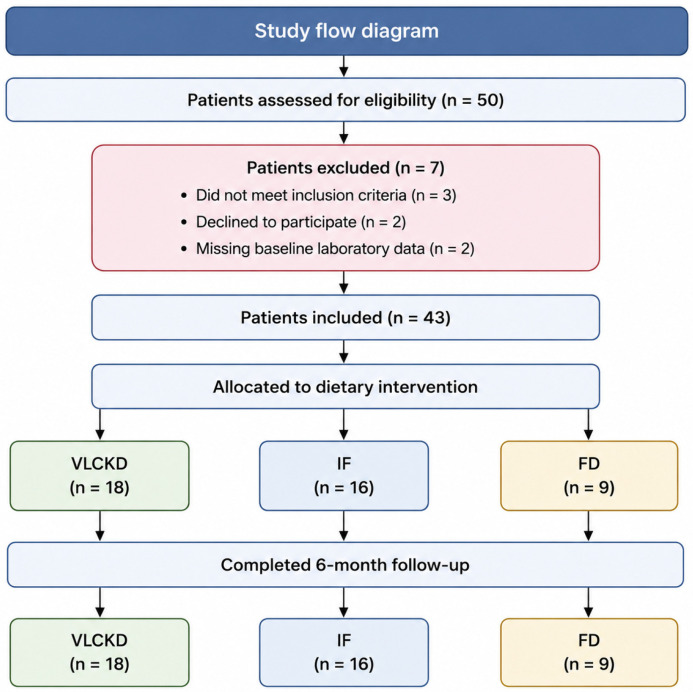
Flowchart illustrating patient screening, exclusion, and allocation to dietary interventions.

Of the initial 50 participants, seven (14%) discontinued the intervention within six months (VLCKD: *n* = 2; IF: *n* = 3; FD: *n* = 2), predominantly due to lack of psychological readiness to adhere to the prescribed dietary regimen despite initial consent. These individuals were excluded from the efficacy analyses but retained for safety evaluations. Efficacy analyses were conducted on a per-protocol basis including participants who completed the 6-month intervention. Participants who discontinued the dietary program were excluded from efficacy analyses due to insufficient exposure to the intervention, while safety monitoring was retained according to the parent protocol. The final numbers included in the efficacy analysis were therefore 18 in the VLCKD group, 16 in the IF group and 9 in the FD group (Figure 1).

All nutritional interventions were individualized and supervised by clinical nutritionists. Each participant underwent an in-person baseline visit, followed by scheduled control visits and weekly telephone contacts to support adherence and address practical issues. Participants were advised to maintain their usual physical activity habits during the study period. Physical activity was not systematically quantified as part of the clinical protocol and was therefore not included as an adjustment variable.

VLCKD was prescribed according to standard protocols with a daily caloric intake of approximately 700–800 kcal/day, and macronutrient distribution characterized by very low carbohydrate intake, moderate protein, and high fat content, as previously described [22,23]. IF was structured as a time-restricted feeding regimen (e.g., 16:8), with defined fasting and feeding windows. Dietary adherence was actively monitored through scheduled clinical visits and structured weekly telephone follow-up, during which participants were systematically asked about compliance with dietary prescriptions, difficulties encountered, and potential deviations from the assigned regimen. Continuous support was provided by clinical nutritionists to enhance adherence. Although detailed quantitative assessment of dietary intake (e.g., food diaries or nutrient analysis) was not performed, adherence was evaluated using a structured clinical approach based on repeated patient interviews and consistency with expected clinical and anthropometric changes. All participants underwent standardized anthropometric and body composition assessments at baseline, after 1 month, and after 6 months from initiation of the dietary protocol. Measurements included height and body weight (recorded in light clothing and without shoes), waist and hip circumferences (measured with a flexible tape in the standing position), and bioelectrical impedance analysis (BIA) using the BIA-DEX^®^ device (Mascaretti Srl, Milan, Italy).

Measurements were performed under standardized conditions (e.g., fasting state, after voiding, and with participants in a supine position, when applicable). All assessments were conducted by trained healthcare professionals to ensure consistency and reduce inter-operator variability. According to the manufacturer’s specifications, the device provides reliable estimates of body composition parameters, with a low measurement error under standardized conditions. Bioelectrical impedance analysis-derived phase angle (PhA) is currently regarded as a robust marker of water distribution, body cell mass, and cellular health and integrity [26]. Additionally, body fat percentage (BFP) and waist–hip ratio (WHR) were assessed at predefined time points. A WHR > 0.80 in women was considered indicative of central adiposity, according to established criteria [27]. BFP was measured and classified according to previously proposed cutoffs, with values of 30–35% generally considered indicative of excess adiposity in women [37].

### 2.4. Laboratory Assessment of Serum Uric Acid

The assessment of UA was planned as part of the original protocol, with the specific aims of evaluating the prevalence of hyperuricemia, using both conventional definitions and emerging CV risk-related thresholds, and potential changes in UA levels in response to distinct nutritional strategies. UA levels (mg/dL) were measured at baseline (T0) and after 6 months of dietary intervention (T6) using standard clinical laboratory methods at our University Hospital. Participants receiving urate-lowering therapy (e.g., allopurinol, febuxostat, uricosuric agents) were not included. Use of medications known to affect UA levels (including diuretics) was documented at baseline and follow-up, and no planned changes were made during the study period. Hyperuricemia was defined according to two different criteria: conventional female-specific cut-off: UA ≥ 6.0 mg/dL [34]; URRAH cut-off: UA ≥ 4.7 mg/dL, associated with increased CV risk even in the absence of overt hyperuricemia [21].

### 2.5. Cardiovascular Assessment

At baseline and at the 6-month follow-up visits, all participants underwent a comprehensive CV assessment as part of routine clinical evaluation at our Hypertension Unit. Assessment of HMOD was performed as part of standard care and included clinical history, physical examination, and review of available instrumental data, such as echocardiographic parameters and carotid ultrasound findings, when indicated. These evaluations were used to define baseline CV risk profile and to contextualize changes in UA within a broader cardiometabolic and vascular framework [25].

#### 2.5.1. Office BP Measurement and Evaluation of Vascular Function

Office BP was measured according to current European Society of Hypertension recommendations [38], using a validated automated oscillometric device [38]. Measurements were obtained in the seated position after at least 5 min of rest, with an appropriately sized cuff, and the average of at least three consecutive readings was recorded. Heart rate was measured simultaneously during BP assessment. In addition to brachial BP measurements, markers of arterial stiffness were assessed through a non-invasive technique, which includes estimation of central BP and pulse pressure, as surrogate markers of large artery stiffness. When available, pulse wave velocity (PWV) or augmentation index (AI@75) oscillometric-derived indices of arterial stiffness were considered, in accordance with standardized protocol adopted at our Hypertension Unit.

#### 2.5.2. Echocardiography and Evaluation of Cardiac Function

Transthoracic echocardiography was performed with the patient in the left lateral decubitus position, in accordance with current recommendations by an experienced echocardiographer using a commercially available ultrasound system (Epiq 7, Philips Ultrasound LLC, Bothell, WA, USA). Left atrial diameter was measured in M-mode from the parasternal long-axis view. Left ventricular (LV) linear dimensions were obtained in M-mode from the parasternal short-axis view at the level of the papillary muscles, and values were averaged over three consecutive cardiac cycles. The following LV parameters were recorded: interventricular septal thickness at end-diastole (IVSd), posterior wall thickness at end-diastole (PWTd), and LV internal diameters at end-diastole (LVIDd) and end-systole (LVIDs). LV ejection fraction (LVEF) was calculated using the Teichholz formula [39]. Epicardial adipose tissue (EAT) thickness was measured perpendicularly to the myocardial surface and identified as the echo-lucent (sono-lucent) space between the outer wall of the right ventricle and the highly echogenic parietal pericardium [39,40,41]. EAT was quantified on parasternal long-axis (LAX) two-dimensional images, using still frames acquired at end-diastole. For each site and view, peak EAT thickness was measured over three cardiac cycles, and the mean of these measurements was used for analysis [40,41,42].

### 2.6. Statistical Analysis

Statistical analyses were performed using SPSS software, version 27.0 (IBM SPSS Statistics, IBM Corp., Armonk, NY, USA). Data normality was assessed using the Shapiro–Wilk test. Baseline characteristics were compared according to UA levels using the conventional female-specific cut-off (≥6.0 mg/dL) and the URRAH threshold (≥4.7 mg/dL), applying Student’s *t* test or Mann–Whitney U test for continuous variables and chi-square or Fisher’s exact test for categorical variables, as appropriate. Within-group changes in UA levels from baseline (T0) to 6 months (T6) were evaluated using paired Student’s *t* test or Wilcoxon signed-rank test. An analysis of covariance (ANCOVA) was performed to assess the independent association between dietary regimen and UA levels at follow-up, adjusting for baseline UA. Given the non-randomized allocation, ANCOVA models were used to mitigate baseline imbalance by adjusting for baseline UA values; residual confounding due to unmeasured lifestyle or pharmacological factors cannot be excluded. Changes in the prevalence of hyperuricemia over time were analyzed using McNemar’s test. Pearson’s correlation was used for normally distributed variables, whereas Spearman’s rank correlation was used for non-normally distributed variables or when normality assumptions were not satisfied. Transitions between CV risk categories defined by the URRAH cut-off (≥4.7 mg/dL) from baseline to follow-up were evaluated within each dietary group, and differences in transition patterns were descriptively compared. Given the nature of the study, no formal sample size calculation was performed, as previously reported [22,23]. All tests were two-sided, with a significance level set at *p* < 0.05.

## 3. Results

### Study Population and Baseline Characteristics

A total of 43 postmenopausal women with essential hypertension and overweight or obesity were included in the present analysis. Eighteen participants were in the VLCKD group, 16 in the IF group, and 9 in the FD group. Participants were stratified according to UA levels using both the conventional female-specific cut-off (≥6.0 mg/dL) and the URRAH threshold (≥4.7 mg/dL). At baseline, women with UA ≥ 6.0 mg/dL had numerically higher body weight (*p* = 0.5), BMI (*p* = 0.5), waist circumference (*p* = 0.7), and BFP (*p* = 0.4) compared with those with UA < 6.0 mg/dL, although no statistically significant differences were observed across most anthropometric or hemodynamic variables. Conversely, EAT was significantly higher in patients with high versus low UA levels (*p* < 0.01). Peripheral and pulse wave-derived BP values were comparable between UA groups, as were indices of left ventricular geometry and diastolic function.

Baseline characteristics across dietary intervention groups (VLCKD, IF, and FD) are detailed in Supplementary Table S1, showing overall comparability between groups despite the smaller sample size in the FD group. Detailed longitudinal anthropometric and metabolic changes are reported in Supplementary Table S2. Similar patterns were observed when participants were stratified using the URRAH cut-off. Women with UA ≥ 4.7 mg/dL exhibited numerically higher BMI (*p* = 0.06), WC (*p* = 0.08), BFP (*p* = 0.4) compared with those below the threshold. Also in this case, EAT was significantly higher in patients with high versus low UA levels (*p* = 0.04) (Table 2).

**Figure 2:**
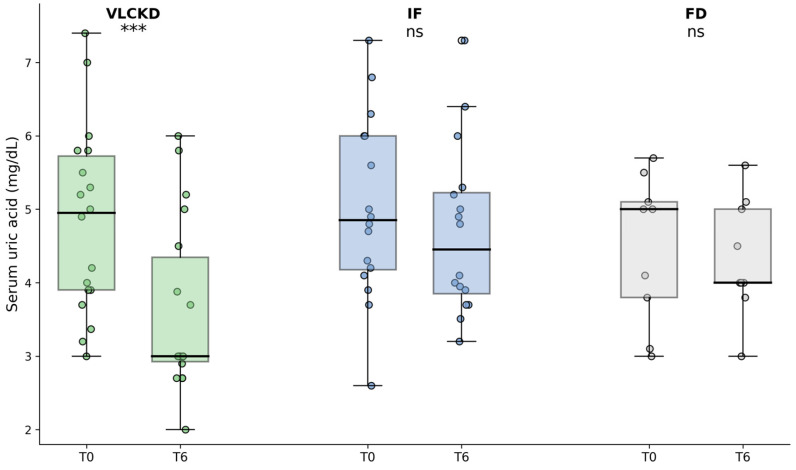
Distribution of serum uric acid (UA) levels at baseline (T0) and 6-month follow-up (T6) according to dietary intervention group. Boxplots represent median and interquartile range values; dots indicate individual participant values. Within-group comparisons were performed using paired statistical analysis. In the figure: *** *p* < 0.001; ns, not significant.

Median and interquartile range UA values at baseline (T0) and 6-month follow-up (T6) for each dietary intervention group are reported in Figure 2.

During follow-up, participants in the VLCKD group showed numerically greater reductions in body weight, BMI, waist circumference, and body fat percentage compared with the IF and FD groups, consistent with the expected metabolic effects of carbohydrate restriction and nutritional ketosis. Detailed longitudinal anthropometric changes are reported in Supplementary Table S2.

Within-group comparisons of UA levels from baseline (T0) to 6 months (T6) revealed distinct effects across dietary regimens. In the VLCKD group (n = 18), a statistically significant reduction in UA levels was observed over time (mean difference = −1.23 mg/dL), corresponding to a relative decrease of approximately 25% from baseline values (*p* < 0.001). These findings should be interpreted in light of the non-randomized design and the relatively small sample size. In contrast, participants following IF (n = 16) showed a smaller and non-statistically significant reduction in UA levels (mean difference −0.33 mg/dL, *p* = 0.18), while no significant change was observed in the FD group (mean difference −0.14 mg/dL, *p* = 0.55).

To account for baseline differences in UA, an analysis of covariance (ANCOVA) model was applied, with UA levels at T6 as the dependent variable and baseline UA levels at T0 and dietary regimen as independent variables. Baseline UA emerged as a strong predictor of UA levels at follow-up (β = 0.63, *p* < 0.001). After adjustment for baseline UA, VLCKD remained independently associated with a statistically significant reduction in UA compared with the FD group (reference category) (β = −0.95 mg/dL, SE = 0.33, *p* = 0.007). No independent effect was observed for the IF regimen (β = 0.02, *p* = 0.96). A detailed summary of the principal statistical analyses, including regression coefficients, confidence intervals, and significance values, is provided in Supplementary Table S3. When hyperuricemia was defined using the conventional female-specific cut-off (≥6.0 mg/dL), a numerical reduction in prevalence was observed from baseline to follow-up in the overall cohort (from 8/43 [19%] to 4/43 [9.3%]), although this change did not reach statistical significance. Conversely, when the URRAH cut-off (≥4.7 mg/dL) was applied, the prevalence of elevated UA decreased significantly over time in the overall population (from 25/43 [58%] at T0 to 15/43 [35%] at T6; McNemar’s exact test *p* = 0.004). Importantly, the use of the URRAH cut-off allowed the identification of a larger proportion of patients at increased CV risk compared with the conventional definition, highlighting its potential utility in detecting subclinical risk. Of note, when adopting this cut-off, the reduction in prevalence was mainly driven by the VLCKD group, in which the proportion of women above the threshold decreased from 10/18 (55.6%) at T0 to 4/18 (22.2%) at T6 (McNemar exact test *p* = 0.031). In the IF and FD groups, smaller and non-statistically significant reductions were observed (IF: 10/16 to 8/16, *p* = 0.50; FD: 5/9 to 3/9, *p* = 0.50). Notably, no transitions from below to above the URRAH threshold occurred in any group.

**Figure 3:**
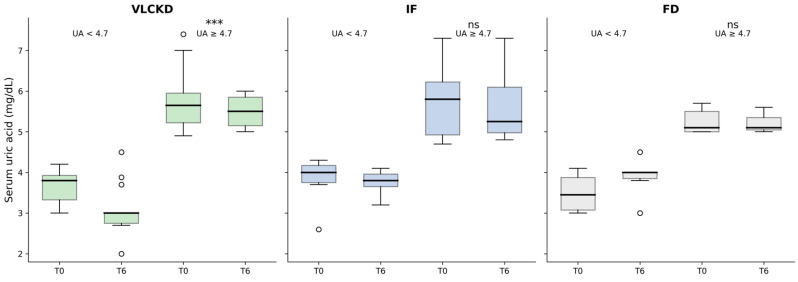
Distribution of serum uric acid (UA) levels according to URRAH-defined cardiovascular risk categories at baseline (T0) and 6-month follow-up (T6) across dietary intervention groups. Boxplots represent median and interquartile range values. Within-group comparisons were performed using paired statistical analysis. In the figure: *** *p* < 0.001; ns, not significant.

At baseline, UA levels were positively correlated with EAT (Spearman’s rho = 0.43; *p* = 0.029), whereas no significant correlations were observed between UA and BFP (rho = 0.24; *p* = 0.115) or PhA (rho = −0.22; *p* = 0.164). EAT was also significantly correlated with BFP (rho = 0.53; *p* = 0.006) and inversely correlated with PhA (rho = −0.54; *p* = 0.004). When participants were further stratified according to baseline CV risk-oriented UA levels (URRAH cut-off ≥4.7 mg/dL) and dietary intervention, a differential pattern of response emerged (Figure 3).

When applying the URRAH cut-off (≥4.7 mg/dL), the reduction in cardiovascular risk prevalence over time was mainly driven by the VLCKD group, in which the proportion of women above the threshold decreased from 10/18 (55.6%) at baseline to 4/18 (22.2%) at follow-up (McNemar exact test *p* = 0.031). In contrast, only modest and non-statistically significant changes were observed in the IF and FD groups (IF: 10/16 to 8/16, *p* = 0.50; FD: 5/9 to 3/9, *p* = 0.50).

Transition analysis confirmed this pattern, showing that 6 out of 18 participants (33.3%) in the VLCKD group shifted from the URRAH+ to the URRAH− category, whereas only 2 out of 16 (12.5%) in the IF group and 2 out of 9 (22.2%) in the FD group experienced a similar improvement. These findings further support the ability of the URRAH-based classification to capture clinically meaningful changes in cardiovascular risk over time. Notably, no transitions from below to above the URRAH threshold were observed in any group. Detailed transition patterns are reported in Supplementary Table S4.

## 4. Discussion

The present real-world study provides novel insights into the relationship among dietary interventions, UA levels, and CV risk in postmenopausal women with essential hypertension and obesity. However, these findings should be interpreted cautiously given the non-randomized design and the relatively small sample size, and therefore should be considered hypothesis-generating. The main findings can be summarized as follows: (i) the use of a lower, CV risk-oriented UA threshold (URRAH cut-off ≥4.7 mg/dL) identified a substantially larger proportion of women at increased risk compared with the conventional definition of hyperuricemia; (ii) among the dietary strategies evaluated, only the VLCKD was associated with a significant and independent reduction in UA levels and in the prevalence of UA-defined CV risk; (iii) elevated UA levels were associated with greater EAT, a recognized marker of adverse cardiometabolic and CV risk.

The greater reduction in UA levels observed in the VLCKD group may be partially explained by the more pronounced reduction in body weight and adiposity-related parameters observed during follow-up. Weight loss is known to improve insulin sensitivity, reduce visceral adiposity, and enhance renal urate handling, all mechanisms potentially contributing to lower UA levels. However, diet-specific metabolic effects may also have played an independent role. In particular, marked carbohydrate restriction and nutritional ketosis may influence UA metabolism through changes in substrate utilization, inflammatory pathways, and insulin-mediated renal urate transport. Therefore, the observed effects are likely multifactorial rather than exclusively attributable to weight reduction alone.

Beyond UA reduction, the dietary interventions were also associated with modifications in several anthropometric and adiposity-related parameters. In particular, participants undergoing VLCKD showed greater reductions in body weight, BMI, WC, and BFP, together with favorable changes in markers associated with visceral adiposity, including EAT. These findings are clinically relevant because visceral fat accumulation and altered body composition are closely linked to inflammation, insulin resistance, endothelial dysfunction, and increased CV risk. Therefore, the observed improvements likely reflect a broader cardiometabolic effect of the dietary interventions beyond UA reduction alone.

A key aspect of this study is the adoption of a novel UA cut-off value, which better reflects emerging evidence that CV risk associated with UA begins at concentrations well below the traditional hyperuricemia threshold. Large epidemiological studies and meta-analyses have consistently demonstrated an independent association between UA and major CV complications, including hypertension, coronary artery disease, heart failure, and CV mortality [41]. In this context, the URRAH study identified sex-specific UA thresholds associated with increased CV risk, proposing a lower cut-off for women that captures subclinical vascular damage In the present cohort, more than half of the participants exceeded the URRAH threshold at baseline; of note, most patients did not meet the conventional definition of hyperuricemia, highlighting the clinical relevance of this risk-oriented approach.

Importantly, at UA levels ≥ 4.7 mg/dL initial signs of HMOD as left ventricular remodeling become evident [21]. Specifically, an increase in relative wall thickness (RWT > 0.42) in the presence of a preserved left ventricular mass index (particularly <95 g/m^2^ in women) identifies a pattern of concentric remodeling, as defined by current echocardiographic recommendations. In parallel, early alterations in diastolic function can be detected, although limited to a single parameter, with a still preserved transmitral E/A ratio (>0.8) [37,43]. Taken together, these findings support the clinical utility of lower UA thresholds for the early recognition of HMOD, potentially facilitating timely therapeutic optimization and improved CV risk stratification, including the risk of major adverse CV events.

Among various HMOD markers, EAT deserves particular attention. EAT is a metabolically active visceral fat depot that exerts paracrine and vasocrine effects on the myocardium and coronary arteries and has been linked to inflammation, insulin resistance, and CV events [44,45,46]. The observed association between higher UA levels and increased EAT thickness supports the hypothesis that UA may reflect early cardiometabolic risk. We recently reported that VLCKD significantly decreased left ventricular mass indexed by height (*p* = 0.01), relative wall thickness (*p* = 0.03), and EAT (*p* < 0.01) compared to baseline values in the same study population. Of note, Spearman analysis revealed a strong correlation between EAT and BFP [24]. Other experimental and clinical studies have shown that intracellular UA may promote oxidative stress, endothelial dysfunction, and inflammatory signaling, mechanisms that are closely related to visceral and epicardial adiposity [24,44]. Importantly, these associations appear to be present even at UA levels below the conventional hyperuricemia threshold, as reported in our analysis, thus reinforcing the concept that “normal” UA values may not be benign in high-risk populations.

From an interventional perspective, the most relevant finding of this study is that only VLCKD resulted in a statistically significant reduction in UA levels over 6 months, an effect that remained significant after adjustment for baseline UA values. In contrast, IF and standard dietary advice were not associated with statistically significant changes in UA levels. The lack of statistically significant changes observed in the intermittent fasting and free diet groups may reflect heterogeneity in adherence, differences in metabolic response, or insufficient statistical power, rather than a true absence of effect. These results are particularly noteworthy given the widespread perception that ketogenic diets may worsen UA levels and increase gout risk. While a transient increase in UA during the early phase of nutritional ketosis has been reported, several clinical studies have demonstrated normalization or reduction in UA levels over time, particularly in association with weight loss and improved insulin sensitivity [22,47,48].

The UA-lowering effect of VLCKD is likely multifactorial. Improvements in insulin resistance, reductions in visceral adiposity, and attenuation of systemic inflammation may collectively enhance renal UA handling and reduce UA production. In addition, ketone bodies—especially β-hydroxybutyrate—have been shown to exert direct anti-inflammatory effects through inhibition of the NLRP3 inflammasome, a pathway implicated in UA-induced vascular inflammation [16]. These mechanisms are consistent with previous evidence from our group demonstrating that a VLCKD improves central (aortic) BP and cardiometabolic risk in postmenopausal women with essential hypertension and obesity suggesting that UA reduction may represent an additional pathway through which VLCKD confers CV benefit [41]. Nevertheless, the long-term sustainability and generalizability of these effects remain uncertain and warrant further investigation in larger and randomized studies.

From a clinical standpoint, our findings may have important implications for CV prevention. Lifestyle modification remains the cornerstone of CV risk reduction, particularly in postmenopausal women, in whom metabolic and inflammatory risk factors tend to cluster [49]. In this real-world setting, VLCKD emerged as the most effective dietary strategy for improving UA-related CV risk, challenging the notion that less restrictive dietary approaches are necessarily safer or more effective. Importantly, the observed benefits were achieved without evidence of adverse effects on renal function or cardiac structure. Overall, these findings highlight the complexity of the relationship between dietary interventions and uric acid metabolism, suggesting that different strategies may exert heterogeneous effects depending on baseline cardiometabolic profile and intervention characteristics.

## 5. Strengths and Limitations

### 5.1. Strengths of the Study

This study has several strengths. First, it provides real-world evidence on the effects of different dietary interventions on UA levels in a clinically relevant and under-investigated population of hypertensive postmenopausal women with obesity. Second, it offers a direct comparison between a VLCKD, IF, and standard dietary advice, allowing a more comprehensive evaluation of their relative impact on UA modulation. Third, the integration of both conventional hyperuricemia thresholds and the URRAH cut-offs represent a novel approach, enabling a more refined assessment of UA-related CV risk.

### 5.2. Limitations of the Study

The present study has several limitations that should be acknowledged. First, the non-randomized design may have introduced selection bias, as dietary allocation was based on clinical and nutritional assessment rather than random assignment. Although this approach reflects real-world clinical practice, it limits causal inference and may have influenced group comparability. In addition, because of the non-randomized design and the relatively small sample size, we could not formally determine whether the observed reduction in UA levels was primarily mediated by weight loss, dietary macronutrient composition, or other metabolic changes associated with the interventions.

Second, the relatively small sample size, particularly in the free diet group, may limit statistical power and increase the risk of type II error, thereby reducing the robustness of between-group comparisons.

Third, dietary adherence was not objectively assessed using validated tools or biomarkers, and was instead monitored through structured clinical follow-up. This may have introduced measurement bias and affected the accuracy of the evaluation of the dietary interventions.

Fourth, physical activity levels and other lifestyle factors were not systematically quantified or controlled, potentially introducing residual confounding. Although patients were on stable pharmacological therapy, the lack of comprehensive multivariable adjustment limits the ability to fully account for all potential confounders.

Finally, the relatively short follow-up period precludes assessment of long-term sustainability and clinical outcomes. In addition, the study population consisted exclusively of hypertensive postmenopausal women, which may limit the generalizability of the findings to other populations.

## 6. Future Perspectives

Future studies should aim to validate these findings in larger randomized controlled trials with adequate statistical power to better define the effects of different dietary strategies on serum uric acid levels and cardiovascular risk. The inclusion of standardized dietary monitoring tools and objective biomarkers of adherence may improve the reliability and reproducibility of results. In addition, comprehensive multivariable models and longitudinal study designs are needed to better establish causal relationships and evaluate long-term clinical outcomes.

Further mechanistic investigations exploring the interplay between uric acid metabolism, visceral adiposity, systemic inflammation, and metabolic regulation may help clarify the biological pathways underlying the observed associations. Finally, extending research to more diverse populations, including men, younger individuals, and patients with different cardiometabolic profiles, will be essential to improve the generalizability and external validity of these findings.

## 7. Conclusions

In this real-world clinical study on postmenopausal women with essential hypertension and obesity, evaluation of UA using the CV risk-oriented URRAH cut-off (≥4.7 mg/dL) identified a substantial burden of subclinical risk—associated with an adverse cardiometabolic profile including increased EAT—and captured meaningful risk reduction over time. Among the lifestyle-based dietary strategies evaluated, VLCKD was the only intervention associated with a significant and independent reduction in UA levels and in the prevalence of UA-defined CV risk over time. These findings challenge prevailing misconceptions regarding ketogenic diets and UA metabolism and support the concept that, when applied within a structured clinical setting, ketogenic dietary interventions may confer meaningful cardiometabolic and vascular benefits. Overall, the present results highlight the importance of a risk-based approach to UA assessment and reinforce the central role of targeted lifestyle interventions in CV prevention. Future studies with larger sample sizes and longer follow-up are warranted to confirm these observations and to further elucidate the role of UA as a modifiable CV risk factor.

## Figures and Tables

**Table 1:** Main characteristics of the dietary interventions.

	VLCKD	IF	FD
Type of intervention	Very low-calorie ketogenic diet	Time-restricted feeding	Standard dietary advice
Caloric intake	700–800 kcal/day	Individualized, comparable to VLCKD over time	Individualized, not strictly controlled
Macronutrient composition	Very low carbohydrates, moderate protein, high fat	No specific macronutrient prescription	Balanced diet according to general recommendations
Meal timing	Multiple structured meals	Defined fasting/feeding window (e.g., 16:8)	No specific timing restriction
Ketosis	Yes	No	No
Individualization	Yes (based on clinical/nutritional profile)	Yes	Yes
Monitoring	Clinical visits + weekly follow-up	Clinical visits + weekly follow-up	Routine clinical follow-up
Objective	Rapid weight loss and metabolic improvement	Metabolic regulation through fasting cycles	Standard-of-care dietary counseling

**Table 2:** General characteristics of the study population stratified using the Uric Acid Right for Heart Health (URRAH) cut-off level of serum uric acid (UA). Data are presented as mean ± standard deviation, median (interquartile range), or number (%), as appropriate. Comparisons between groups were performed using ANOVA, Kruskal–Wallis test, or chi-square test, as appropriate.

Parameters	UA < 4.7 mg/dL	UA ≥ 4.7 mg/dL	*p* Value
	(*n* = 18)	(*n* = 25)	
Age (years)	60.89 ± 5.31	60.64 ± 4.61	0.87
Body weight (kg)	79.52 ± 11.82	86.06 ± 16.09	0.13
Height (m)	1.60 ± 0.09	1.58 ± 0.06	0.42
BMI (kg/m^2^)	31.00 ± 4.55	34.40 ± 6.50	0.05
Waist circumference (cm)	93.64 ± 8.29	99.55 ± 13.44	0.08
Hip circumference (cm)	111.94 ± 12.64	114.11 ± 12.62	0.58
Waist-to-hip ratio	0.84 ± 0.07	0.87 ± 0.07	0.17
BFP (%)	38.39 ± 7.35	42.38 ± 8.07	0.10
EAT (mm)	6.25 ± 0.93	6.97 ± 1.13	0.04
Extracellular water (%)	48.62 ± 4.52	50.29 ± 5.06	0.26
Intracellular water (%)	51.38 ± 4.52	49.71 ± 5.06	0.26
Total body water (%)	44.57 ± 5.29	42.13 ± 5.60	0.15
Systolic BP (mmHg)	136.72 ± 17.04	134.40 ± 16.17	0.65
Diastolic BP (mmHg)	86.11 ± 10.69	86.52 ± 10.23	0.90
Pulse pressure (mmHg)	50.61 ± 13.63	49.80 ± 12.38	0.84
AST (U/L)	21.94 ± 4.80	23.78 ± 6.97	0.31
ALT (U/L)	23.44 ± 6.71	27.16 ± 17.50	0.34
Serum creatinine (mg/dL)	0.8 ± 0.22	0.8 ± 0.13	0.99
eGFR (ml/min/1.73 m^2^)	70 ± 0.44	68 ± 0.91	0.28
LV end-diastolic diameter (mm)	46.56 ± 3.15	46.32 ± 3.91	0.82
LV end-systolic diameter (mm)	29.50 ± 3.45	28.96 ± 3.95	0.63
RWT	0.42 ± 0.04	0.44 ± 0.06	0.19
PWV (m/s)	8.42 ± 0.51	8.90 ± 0.68	0.08

## Data Availability

The original contributions presented in this study are included in the article/Supplementary Material. Further inquiries can be directed to the corresponding author.

## Supplementary Materials

The following supporting information can be downloaded at: https://www.mdpi.com/article/10.3390/nu18121912/s1, Table S1: Baseline characteristics according to serum uric acid cut-offs; Table S2: Longitudinal anthropometric and metabolic changes according to dietary intervention; Table S3: Summary of principal statistical analyses; Table S4: Transitions of URRAH-defined cardiovascular risk status.

## Abbreviations

AI@75	augmentation index normalized to a heart rate of 75 bpm
ALT	alanine aminotransferase
ANCOVA	analysis of covariance
AST	aspartate aminotransferase
Β	regression coefficient
BFP	body fat percentage
BIA	bioelectrical impedance analysis
BMI	body mass index
BP	blood pressure
CV	cardiovascular
EAT	epicardial adipose tissue
eGFR	estimated glomerular filtration rate
FD	free diet
HMOD	hypertension-mediated organ damage
IF	intermittent fasting
IQR	interquartile range
KD	ketogenic diet
LV	left ventricular
LVEF	left ventricular ejection fraction
PWV	pulse wave velocity
rho (ρ)	Spearman’s correlation coefficient
ROS	reactive oxygen species
RWT	relative wall thickness
SE	standard error
T0	baseline
T6	6-month follow-up
UA	uric acid
URRAH	Uric Acid Right for Heart Health
VLCKD	very low-calorie ketogenic diet
WHR	waist-to-hip ratio
XO	xanthine oxidase
