# Supplementary material for: Serum Uric Acid as a Cardiovascular Risk Marker: Differential Effects of Ketogenic Diet and Intermittent Fasting in Postmenopausal Women
Source: Nutrients. 2026 Jun 12;18(12):1912. doi: 10.3390/nu18121912 (PMC13305224; doi:10.3390/nu18121912)
Supplement: Supplementary file 1 [file nutrients-18-01912-s001.zip › nutrients-4267578-supplementary.pdf]

Supplementary Table S1.
Baseline demographic, anthropometric, metabolic, vascular, and echocardiographic characteristics according to dietary intervention group and uric acid thresholds.

**Characteristics**	**VLCKD**			**IF**		
	**UA <6.0 mg/dL**	**UA ≥6.0 mg/dL**	**P value**	**UA <6.0 mg/dL**	**UA ≥6.0 mg/dL**	**p value**
Age (years)	60.87 ± 3.72	62.00 ± 5.29	0.75	60.64 ± 4.43	57.40 ± 6.99	0.38
Body weight (kg)	83.09 ± 13.45	94.40 ± 20.54	0.44	83.36 ± 16.18	83.02 ± 17.83	0.97
Height (m)	1.60 ± 0.09	1.61 ± 0.03	0.77	1.60 ± 0.05	1.59 ± 0.05	0.72
BMI (kg/m²)	32.56 ± 6.00	36.61 ± 9.25	0.53	32.42 ± 5.67	33.11 ± 7.40	0.85
Waist circumference (cm)	96.24 ± 11.20	105.00 ± 12.68	0.35	98.31 ± 11.27	94.76 ± 17.33	0.69
Hip circumference (cm)	113.67 ± 11.97	117.00 ± 14.40	0.73	111.15 ± 12.36	114.44 ± 15.57	0.69
Waist-to-hip ratio	0.85 ± 0.05	0.90 ± 0.03	0.07	0.89 ± 0.05	0.83 ± 0.10	0.26
Fat mass (%)	40.79 ± 9.54	41.90 ± 3.75	0.74	39.84 ± 7.85	42.72 ± 7.90	0.51
Epicardial adipose tissue (mm)	6.88 ± 0.83	7.50 ± 0.71	0.26	6.30 ± 1.43	7.25 ± 0.51	0.07
Extracellular water (%)	49.62 ± 6.12	48.90 ± 4.11	0.81	49.05 ± 4.39	51.00 ± 3.84	0.39
Intracellular water (%)	50.38 ± 6.12	51.10 ± 4.11	0.81	50.95 ± 4.39	49.00 ± 3.84	0.39
Total body water (%)	43.39 ± 6.63	43.00 ± 1.47	0.84	43.38 ± 5.68	41.94 ± 5.19	0.63
Systolic BP (mmHg)	132.60 ± 16.97	136.00 ± 20.07	0.80	138.55 ± 15.88	135.00 ± 12.27	0.63
Diastolic BP (mmHg)	82.27 ± 10.66	86.33 ± 8.02	0.49	87.91 ± 8.47	87.80 ± 5.89	0.97
Pulse pressure (mmHg)	50.33 ± 15.78	49.67 ± 15.01	0.94	50.64 ± 11.02	56.80 ± 13.46	0.40
AST (U/L)	20.93 ± 6.58	28.67 ± 10.41	0.32	23.45 ± 5.11	20.10 ± 4.04	0.18
ALT (U/L)	25.47 ± 21.40	36.00 ± 14.93	0.36	25.73 ± 5.78	20.40 ± 9.86	0.30
UA (mg/dL)	4.45 ± 0.96	6.80 ± 0.72	0.01	4.35 ± 0.80	6.48 ± 0.56	0.001
LV end-diastolic diameter (mm)	47.67 ± 3.04	48.00 ± 5.20	0.92	47.09 ± 3.27	43.60 ± 2.88	0.07

LV end-systolic diameter (mm)	29.13 ± 3.44	31.67 ± 6.03	0.54	29.64 ± 3.20	29.60 ± 5.22	0.98
RWT	0.42 ± 0.04	0.44 ± 0.06	0.62	0.42 ± 0.07	0.48 ± 0.06	0.11
LV mass/height (g/m)	89.63 ± 14.09	96.11 ± 11.09	0.43	94.45 ± 16.61	88.96 ± 16.21	0.55
LV mass indexed to BSA (g/m2)	74.95 ± 11.46	75.78 ± 7.84	0.88	79.72 ± 15.94	74.50 ± 14.79	0.54
LV mass indexed to height^2.7 (g/m^2.7)	40.57 ± 7.77	42.83 ± 5.52	0.58	42.35 ± 6.97	40.94 ± 8.90	0.70
Left atrial volume index (mL/m2)	24.62 ± 2.33	31.90 ± 15.38	0.49	27.87 ± 6.03	23.59 ± 5.29	0.18
Mitral E-wave velocity (cm/s)	62.53 ± 12.05	50.67 ± 16.92	0.34	49.73 ± 14.36	67.80 ± 20.19	0.22
Deceleration time (ms)	152.40 ± 32.57	146.33 ± 17.56	0.66	149.55 ± 19.33	178.60 ± 43.18	0.21
E/A ratio	0.82 ± 0.18	0.74 ± 0.06	0.19	0.79 ± 0.19	0.93 ± 0.29	0.36
TDI e′ velocity medial (cm/s)	8.93 ± 1.15	8.50 ± 1.32	0.64	9.09 ± 1.45	9.78 ± 1.28	0.36
TDI e′ velocity lateral (cm/s)	8.73 ± 1.03	8.33 ± 1.15	0.61	8.27 ± 1.90	9.00 ± 1.00	0.33

Supplementary Table S2

Longitudinal anthropometric and metabolic changes from baseline (T0) to 6-month follow-up (T6) according to dietary intervention group.

**Parameters**	**VLCKD T0**	**VLCKD T6**	**IF T0**	**IF T6**	**FD T0**	**FD T6**
Body weight (kg)	84.98 ± 14.74	74.43 ± 14.52	83.26 ± 16.10	78.66 ± 15.93	80.12 ± 12.80	79.24 ± 13.76
BMI (kg/m²)	33.23 ± 6.49	29.10 ± 6.21	32.64 ± 6.01	30.86 ± 6.04	33.06 ± 5.32	32.68 ± 5.70
PhA	5.95 ± 0.78	6.34 ± 0.53	5.64 ± 0.57	5.99 ± 0.52	5.89 ± 0.63	5.67 ± 0.55
BFP (%)	40.97 ± 8.76	32.35 ± 8.67	40.74 ± 7.72	37.39 ± 7.00	40.13 ± 7.45	39.87 ± 7.70
EAT (mm)	7.00 ± 0.82	7.00 ± 0.82	6.62 ± 1.26	5.39 ± 1.57	6.08 ± 1.14	6.08 ± 1.14
UA (mg/dL)	4.84 ± 1.27	3.62 ± 1.18	5.01 ± 1.25	4.68 ± 1.15	4.48 ± 1.01	4.33 ± 0.80

Supplementary Table S3.

Summary of the principal statistical analyses performed in the study. Within-group comparisons were performed using paired Student's t-test or Wilcoxon signed-rank test, as appropriate. Correlations were assessed using Spearman's rank correlation coefficient. ANCOVA models were adjusted for baseline serum uric acid values. β values indicate the direction and magnitude of association in ANCOVA models. Positive rho (ρ) values indicate direct correlations, whereas negative rho values indicate inverse correlations. Higher absolute rho values indicate stronger associations. Statistical significance was defined as $p < 0.05$

**Comparison**	**Statistical test**	**Main results**	**p value**
**Longitudinal changes**			
VLCKD: UA T0 vs T6	Paired t-test	ΔUA = −1.23 mg/dL	< 0.001
IF: UA T0 vs T6	Paired t-test	ΔUA = −0.33 mg/dL	0.18
FD: UA T0 vs T6	Paired t-test	ΔUA = −0.14 mg/dL	0.55
**ANCOVA**			
Baseline UA predicting follow-up UA	ANCOVA	β = 0.63	< 0.001
VLCKD vs FD (reference category)	ANCOVA	β = −0.95 mg/dL; SE = 0.33	0.007
IF vs FD (reference category)	ANCOVA	β = 0.02	0.96
**Prevalence analysis**			
Overall: URRAH prevalence T0 vs T6	McNemar's exact test	58% → 35%	0.004
VLCKD: URRAH prevalence T0 vs T6	McNemar's exact test	55.6% → 22.2%	0.031
**Correlations**			
UA vs EAT	Spearman correlation	rho = 0.43	0.029
UA vs BFP	Spearman correlation	rho = 0.24	0.115
UA vs PhA	Spearman correlation	rho = −0.22	0.164
EAT vs BFP	Spearman correlation	rho = 0.53	0.006
EAT vs PhA	Spearman correlation	rho = −0.54	0.004

Supplementary Table S4

Transitions of URRAH-defined cardiovascular risk status (UA ≥4.7 mg/dL) from baseline (T0) to 6 months (T6) according to dietary intervention.

URRAH cut-off defined as UA ≥4.7 mg/dL. No transitions from below to above the URRAH threshold were observed in any dietary group.

**VLCKD group (n = 18)**		
URRAH status	T6 <4.7 mg/dL	T6 ≥4.7 mg/dL
T0 <4.7 mg/dL	8	0
T0 ≥4.7 mg/dL	6	4

McNemar exact p = 0.031

**IF group (n = 16)**		
URRAH status	T6 <4.7 mg/dL	T6 ≥4.7 mg/dL
T0 <4.7 mg/dL	6	0
T0 ≥4.7 mg/dL	2	8

McNemar exact p = 0.50

**FD group (n = 9)**		
URRAH status	T6 <4.7 mg/dL	T6 ≥4.7 mg/dL
T0 <4.7 mg/dL	4	0
T0 ≥4.7 mg/dL	2	3

McNemar exact p = 0.50
